# Breakfast Frequency and Composition in a Group of Polish Children Aged 7–10 Years

**DOI:** 10.3390/nu13072241

**Published:** 2021-06-29

**Authors:** Anna Kawalec, Krystyna Pawlas

**Affiliations:** Department of Hygiene, Wroclaw Medical University, 50-367 Wroclaw, Poland; krystyna.pawlas@umed.wroc.pl

**Keywords:** breakfast composition, breakfast pattern, school-age children, lifestyle

## Abstract

Breakfast is considered one of the crucial elements of a healthy diet. Most studies evaluate breakfast consumption with the risk of obesity and other health effects. Less attention is paid to the evaluation of breakfast composition and patterns. Thus, this study aimed to describe the most frequently observed breakfast patterns and to assess breakfast composition and quality in a group of Polish early school-age children. The cross-sectional survey study was conducted in school years 2017/2018 and 2018/2019. Information regarding breakfast was obtained with the use of an original paper-based weekly observation diary, and breakfast quality was assessed with a special scoring designed to be used together with the diary. In total, 223 schoolchildren of the second and third grades participated in the study, and 200 diaries were analyzed. More than ¾ of the participants consumed breakfast every day. Nearly 68% of meals were classified as well balanced, but only 16.5% of children eat a well-balanced breakfast every day. The number of children who usually (≥5 times per week) eat a sandwich for breakfast was 94 (47%), and that of those who habitually eat cereal and milk or porridge was 29 (14.5%). Only 7% of children consumed fruit or vegetables for breakfast daily, and 26.5% never eat fruit or vegetables for breakfast. Concluding, most children eat breakfast regularly, but the meal composition and quality might be improved.

## 1. Introduction

The dietary pattern is an essential factor influencing the general health and well-being of an individual. Breakfast has been labeled the most important meal of the day and is considered one of the crucial elements of a healthy diet. Proper nutrition, including regular breakfast consumption, has a great impact on children’s growth and development [1,2].

Several studies indicate that eating breakfast improves cognitive function, particularly attention and episodic memory, and may contribute to learning and academic performance in children and adolescents [3,4,5,6]. Additionally, the relationship between breakfast consumption and disease prevalence has been studied extensively. It is suggested that skipping breakfast is associated with unfavorable plasma cholesterol levels and increases the risk of arteriosclerosis, cardiovascular diseases, type 2 diabetes, metabolic syndrome, and obesity [7,8,9,10,11]. Children who consume breakfast regularly are more likely to have favorable macro- and micro-nutrient intake, including higher intake of dietary fiber and total carbohydrates, and lower total fats and cholesterol [12,13], while skipping breakfast has been associated with a lower likelihood of meeting recommended nutrient intakes [12,13].

It is estimated that only about 65% of Polish school-age children eat breakfast every day, and the problem of skipping breakfast increases with age [14]. Noteworthy, having low meal frequencies in early adolescence predicts low meal frequencies in late adolescence and early adulthood [15]. These observations underline the need for regular breakfast consumption promotion and shaping health behaviors among school children.

Most studies evaluate breakfast consumption rates with the risk of overweight or obesity and other health effects. Although breakfast patterns and the composition of children and teenagers from different countries and regions have been assessed previously [16,17,18], the number of studies focusing on breakfast composition among Polish children is rather limited. It should be underlined that there are significant cultural and behavioral differences in the diet of given populations and that dietary habits differ according to age. Therefore, we cannot simply extrapolate the results of studies regarding breakfast composition conducted in other countries and other age groups to the population of Polish early school-age children. Thus, the aim of this study was the description of the most frequently observed breakfast patterns and the assessment of breakfast composition and quality among Polish early school-age children. As evidence shows that regular breakfast consumption decreases with age, we decided to conduct this study among children aged 8 to 9 years, as this age group seems to be the target group for tailored health promotion programs.

## 2. Materials and Methods

### 2.1. Participants and Settings

This study was approved by the Bioethics Committee of Wroclaw Medical University. The study was conducted in school years 2017/2018 and 2018/2019. Inclusion criteria for schools were location (city Wroclaw) and the permission of school principals for the school’s participation in the research study. We invited 46 randomly selected schools out of 81 elementary schools of Wroclaw. In total, children from the 2nd and 3rd grades of six elementary schools were invited to participate in the study.

### 2.2. Study Design

Detailed information regarding breakfast consumption and its composition was obtained with the use of a special paper-based food record. The diary ‘Seven days for my health’ authored by Domenico Tiso is an interactive tool to assess the lifestyle of school children aged 6 to 11 years. It was used in several studies in Italy and showed to be effective and adequate for the qualitative assessment of nutrients and food intake [19,20]. The innovation of the diary is the presentation of the nutritional section as a coloring booklet. The child indicates the kind of food consumed for each meal by coloring the picture of the food and drink item (if the food type is available in the diary) or drawing and coloring the food in a provided space. The diary is filled and colored immediately after a meal with the supervision of parents. Each diary was administered with a short written instruction on how to correctly compile the diary.

The diary was translated and adapted for use among the group of Polish early school-age children. The observation lasted seven consecutive days (from Monday to Sunday). On the diary’s cover, there was a space for the parents to fill in the child’s demographic data, such as gender and birth date. To complete the observation for breakfast, children indicated if they had breakfast and could either color or circle the food item they just consumed, depending on the amount of time they had. For each day of the week, we analyzed if the child ate breakfast, and if yes, what was eaten and drunk. Beverage item pictures for breakfast included: water, tea, milk, cacao, juice in a carton or bottle, fresh cold-pressed juice, sweet drink. Food item pictures for breakfast included: sandwich (with cheese, cottage cheese, ham or sausage, jam, chocolate, other), cereals, sweet roll (bun), yogurt, eggs, fruit, vegetables. If the child used additional space to draw and write what was eaten and drunk for breakfast, this item was treated as ‘other’ in the analysis of meal composition.

Completed diaries were collected and evaluated for completeness and accuracy by one person, in order to avoid bias due to differences in the assessment.

### 2.3. Data Analysis and Presentation

Breakfast was considered as consuming solid food with or without beverages at the first eating episode per day. Breakfast frequency, composition, and content of different food and beverage items in each meal were analyzed for seven days of observation for each child. We assessed how many children skipped breakfast or consumed only liquids during the observation period, and if these subgroups differ according to age or gender.

The qualitative assessment of breakfast composition was conducted using a specially designed score. The scoring criteria were designed by an Italian research group to use together with the diary [20]. The usage of this score to assess if breakfast is a well-balanced meal is supported by the Polish recommendations of a healthy diet and meal composition presented in the form of a healthy eating plate [21]. Breakfast composition was rated according to the content of water (+1), carbohydrates (+1), proteins or dairy products (+1), fiber (+1), vitamins/minerals (+1), and free sugars (−1), assuming a well-balanced breakfast if scoring ≥3 points (each point in a different category) and containing a source of proteins or dairy products and carbohydrates. The classification of each food and drink item according to this score is presented in Supplementary Table S1.

Due to differences in the frequency of consumption of a well-balanced breakfast, the study group was divided into two subgroups: children who consumed a well-balanced breakfast five or more times per week and those who consumed a well-balanced breakfast four or less times per week. Demographic characteristics, consumption frequency of different food products, and content of food and beverage items in meals for both groups were compared.

In addition, we tried to describe the most frequently observed breakfast patterns. In particular, we focused on sandwich or cereal and milk consumption frequency. If the child reported eating a ‘sandwich’ or ‘cereals and milk’ for breakfast five or more days per week, we characterized this pattern as ‘usually eats sandwich’ or ‘usually eats cereals and milk’, respectively. Additionally, for each child, we assessed how many breakfasts during the week included a portion of fruit or vegetables.

The child’s age was calculated using the birth date written by the parent and the start date of the seven-day observation.

In general, 223 children completed observations for 1496 days. However, for further analysis, we included 1400 observations from 200 fully completed diaries. Analyzed data consisted of 1304 meals containing solid food with or without beverages reported by 200 children (Table 1).

### 2.4. Statistical Analysis

Descriptive statistics are presented as the mean (M) or median (Me) and standard deviation (SD) from the mean. Qualitative comparison of variables between subgroups was conducted with the use of the chi-square test. The significance of differences in the observed frequency of consumption was checked with Student’s t-test. The significance level was assumed as *p* < 0.05. Statistical analyses were performed using the spreadsheet LibreOffice Calc which is a component of the LibreOffice software package.

## 3. Results

### 3.1. Sample Size and Demographic

In total, 223 children aged from 7 to 10 years (mean age 8.7 ± 0.5 years) participated in this study. In this group, 200 diaries (89.69%) were fully compiled and contained observations for breakfast for seven consecutive days reported by 114 girls (57%) and 86 boys (43%).

### 3.2. Breakfast Regular Consumption and Breakfast Skipping

**Figure 1:**
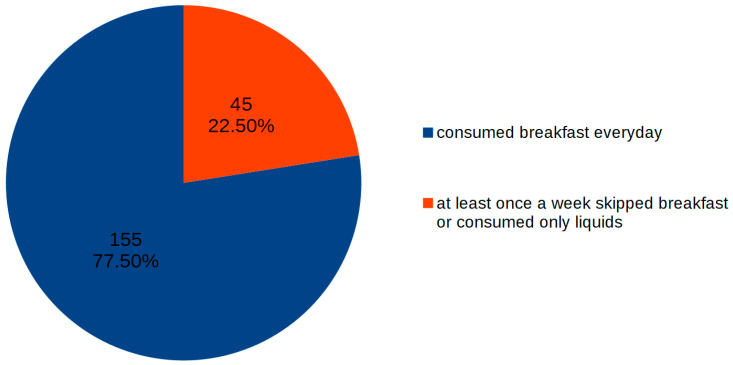
Regularity of breakfast consumption during a week in the group of 200 children.

According to the fully completed diaries, more than ¾ of the participants consumed breakfast every day. However, 45 children (22.5%) admitted that they did not consume solid food for breakfast at least once a week, including nine children who skipped breakfast at least once a week (Figure 1). We observed no difference between everyday breakfast consumption and the child’s sex or age (Table 2).

### 3.3. Breakfast Composition—Qualitative Assessment

**Figure 2:**
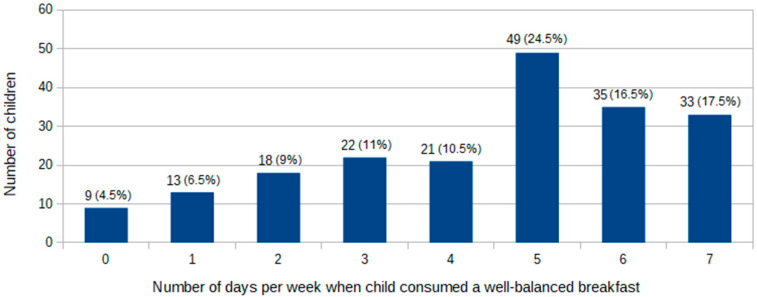
Frequency of the consumption of a well-balanced breakfast during a week.

Among 1304 morning meals containing solid food, 1006 (77.14%) were scored ≥ 3 points or above, and 885 (67.87%) were classified as well-balanced (i.e., obtained ≥ 3 points and contained a source of protein or dairy products and carbohydrates). However, only 33 children consumed a well-balanced breakfast every day (Figure 2). According to differences in the frequency of consumption of a well-balanced breakfast, the study group was divided into two subgroups: children who consumed a well-balanced breakfast five or more times per week and those who consumed a well-balanced breakfast four or less times per week. The demographic characteristics of both groups are presented in Table 2.

We observed a significant difference between the everyday consumption of a well-balanced breakfast during school days and weekend days (Table 3).

**Figure 3:**
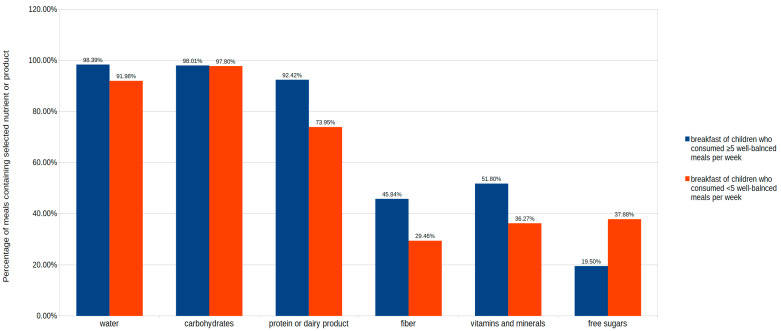
A qualitative assessment of breakfast composition in groups different by frequency of consumption of well-balanced breakfast.

Analysis of 1383 observations showed that, in total, more than 92% of analyzed meals contained a source of carbohydrates and more than 80% a source of protein or a dairy product. Differences in breakfast composition and the content of nutrients in a group of children who usually consume well-balanced meals and those who consume well-balanced meals less frequently are presented in Figure 3.

In addition, meals were analyzed according to the content of selected beverage and food items and their consumption frequency for both subgroups (Table 4, Table 5 and Table 6). Most often, children drink tea, water, or milk and eat sandwiches or cereal.

### 3.4. Breakfast Patterns

According to the analysis of the frequency of different product consumption, we attempted to describe the most frequently observed breakfast patterns. In particular, we focused on sandwich or cereal and milk consumption frequency. The dominant breakfast pattern in the study group was eating a sandwich for breakfast (most frequently with ham or cheese). The number of children who usually (≥5 times per week) eat a sandwich for the morning meal was 94 (47%). The number of children who usually (≥5 times per week) eat cereals and milk or porridge for breakfast was 29 (14.5%).

**Figure 4:**
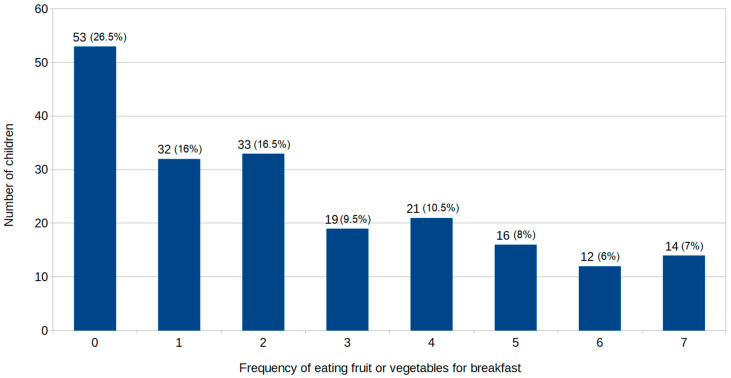
Frequency of fruit and vegetable consumption for breakfast.

In Poland, the National Food and Nutrition Institute recommends that vegetables and fruit dominate the diet of adults and children and should constitute half of what is eaten. It is advised to eat a variety of vegetables and fruits as often and as much as possible, with the proportion of more vegetables than fruit, preferably by including a portion of a fruit or vegetable in every meal [21,22]. Therefore, we assessed how often children eat breakfast that is accompanied by fruit or vegetables. Only 7% consumed fruit or vegetables for breakfast every day, and 26.5% never eat fruit or vegetables for breakfast (Figure 4).

## 4. Discussion

Regular breakfast consumption is one of the key elements of a healthy diet. This study extends current knowledge about dominant breakfast patterns and breakfast composition among Polish early school-age children.

Our findings show the percentage of children who consume breakfast every day to be 77.5%. This is in line with other studies conducted in Poland which estimate the percentage of children who eat breakfast every day to be 81% among 8-year-olds [23] and 87% among a group of 6–13-year-olds [24]. Similarly, Galczak-Kondraciuk et al. reported that 77% of children aged 7–12 years had breakfast before going to school; however, these data are for a one-day observation [25]. Wadolowska et al. observed that 17.4% of teenagers aged 11–13 years frequently skipped breakfast and identified that age over 12 years is one of the predictors of missing breakfast [9]. Another study conducted among adolescents showed that breakfast was not consumed regularly by 12.5% of girls and 9.3% of examined boys [26]. 

The breakfast consumption rates and meal composition vary significantly between geographical regions and countries, mainly due to cultural and socioeconomic determinants. Data regarding breakfast habits among young children aged 4–8 years in Cyprus showed that 63.8% of boys and 62.7% of girls reported eating breakfast daily [27]. Similarly, approximately 63.3% of British children aged 9–10 years consume breakfast daily [28]. According to recent findings from the 2017/2018 Health Behavior in School-aged Children (HBSC) survey, the lowest overall levels of daily breakfast consumption among schoolchildren were observed in Central European countries, while the highest were observed in the Netherlands [14]. Data from the 2017/2018 HBSC survey showed a decline in the percentage of Polish 11-year-olds who eat breakfast every school day from 2014 to 2018, from 70% among girls and 72% among boys to 65% and 67%, respectively [14].

Although the downward trend in regular breakfast eating is observed among Polish teenagers, it is unclear if this unfavorable change is present among early school-age children because of a lack of studies in this particular age group. However, several studies worldwide revealed that children become less likely to consume breakfast regularly when entering the critical pubescent growth period. For instance, a prevalence of breakfast consumption of 80.4% was observed among Jordanian school children aged 6–14 years, but the percentage of children eating breakfast every day was only 49.7%, and those eating breakfast 5–6 days per week was 11.5%. The occurrence of missing breakfast increased with age, from nearly 10% among the 6–9 years age group to almost 30% among the 9–12 years age group [29]. Likewise, only 62.6% of Turkish children from 6 to 18 years old had breakfast every day, and the percentage of children eating breakfast daily also significantly decreased with age (79.1% at 6–11 vs. 52.1% at 12–18 years) [30].

The most prevalent breakfast pattern in the study group was eating a sandwich, which was observed among 47% of the children, whereas 14% of the children usually consumed ready-to-eat cereals and milk or porridge. Nearly 68% of meals were classified as well balanced, and about 37.5% of meals contained a portion of fruit or vegetables.

This is partially in line with Zielińska et al.’s findings, who reported that 53% of children aged 6 to 13 years old eat sandwiches for breakfast (28% with vegetables of fruit, and 25% without vegetables or fruit), 32% eat ready-to-eat cereal, and 9% eat porridge or milk soup [24]. The disparity might be due to the different age group and type of questionnaire used (7-day observation diary vs. single choice of frequency category regarding foodstuffs and dishes eaten most often). The composition of breakfast was also evaluated among Polish teenagers aged 12 to 17 years old, which showed that nearly all meals contained grain products, while dairy products were consumed by 46.4% of respondents, and vegetables and fruit by 20.3% and 6.6%, respectively [31]. However, only approximately 13.8% of morning meals were assessed as well balanced [31].

According to the systematic review of 24 studies regarding breakfast quality, content, and context, the most commonly consumed breakfast items were the ready-to-eat cereal and dairy foods, fruit and fruit juice, and bread [32]. However, there is a large discrepancy between countries and regions. Similar to our findings, a study conducted in German children and adolescents showed that 62% of breakfasts were bread meals, and 21% were ready-to-eat cereals, while 1% of breakfast meals contained only beverages. In addition, only 24% of bread or ready-to-eat cereal meals contained fruit or vegetables or dairy products [33]. Likewise, bread, butter, and milk were the most frequently consumed products by children and teenagers in the Netherlands [34]. In contrast, in Portugal, the most frequent food items that children eat for breakfast were dairy products and ready-to-eat cereal, and 53.5% of meals were a combination of milk and cereal, or milk, cereal, and bread. In this study, fruit consumption was reported by 9.8% of children, with 4.8% of children and adolescents having a qualitative and complete breakfast (bread/cereal, dairy products, and fruit) [35]. In a group of Italian children aged 6 to 11 years old, the most often eaten food items for breakfast were milk (38.9%) and biscuits (34.9%), and only 9% of children consumed a well-balanced breakfast every day during the week [36]. In a French study, the most frequently eaten food items were: sweets, consumed by 67.7% of children, bread (46.8%), and butter (41.5%). Almost half of the children drunk flavored milk, while ready-to-eat cereals, which are not a traditional breakfast food in France, were consumed by 28.1% of children [16].

Although there are large differences in the most frequently chosen food items for breakfast, the majority of meals include grain products, while fruit or vegetables are consumed less frequently. According to the Polish National Food and Nutrition Institute guidelines, preferably each main meal should include a portion of fruit or vegetables [21]. Our findings showed that only 7% of children met this recommendation and consumed fruit or vegetables for breakfast every day, while 26.5% never eat fruit or vegetables for breakfast.

A strength of the current study was the use of a weekly observation diary to measure breakfast consumption and its quality. The majority of previous studies assessed the frequency of skipping breakfast and rarely evaluated its composition. As the observation lasted seven days, it enabled us to describe the most frequent breakfast patterns. Possible limitations should be listed as well. The Polish version of the ‘Seven days for my health’ diary has not been validated. The results are based on self-reported data and thus depend on subject memory. In addition, recording errors may occur. However, we advised that the child fills in the diary shortly after a meal and under the supervision of a caregiver to minimize potential bias. The diary was designed to be simple and easy and included only the main group of products, without subdivision to white bread or whole-grain bread, natural or sweet yogurt, etc. Although these limitations exist, the data reported in the diaries enabled a general qualitative assessment of meal composition [20].

A review of national policies and health campaigns promoting the consumption of breakfast in Europe placed Poland among a group of countries in which governments need to increase their focus on breakfast consumption as part of a healthy dietary pattern and better inform the general public on the scientifically proven benefits of positive breakfast behavior throughout one’s lifetime [36]. Recently, many strategies promoting a healthy lifestyle were implemented, including creating the National Center for Nutrition Education, awareness campaigns addressed to children and families, and programs promoting healthy dietary choices in almost all elementary schools, for instance, ‘Milk at school’ or ‘Vegetables and fruit at school’.

Nonetheless, our findings have important implications for the field of health promotion. Efforts should be aimed not only towards increasing regular breakfast consumption but also towards improving breakfast composition and quality. There is a need for nutritional education for school children and their parents. Breakfast is a meal usually eaten at home, and the diet of school-age children is primarily determined by parental decisions.

## 5. Conclusions

This study showed that only about ¾ of early school-age children eat breakfast regularly, and 2/3 of them usually eat a well-balanced breakfast. This situation is not satisfactory and underlines the need for actions to improve regular breakfast consumption and quality. These findings might be seen as a potential starting point for tailored intervention programs, including awareness campaigns addressed to children and their parents.

## Figures and Tables

**Table 1:** Observations for breakfast consumption derived from 200 fully completed diaries.

Type of Observation for Breakfast Consumption	Number of Observations	Percentage
Solid food with or without beverage	1304	93.14%
Only liquids	79	5.64%
No breakfast	17	1.21%
Total	1400	100%

**Table 2:** Demographic characteristics of children different in everyday breakfast consumption and in the frequency of consumption of well-balanced breakfast.

Variables	Children Who Consumed Breakfast Every Day during the Week		Children Who Skipped Breakfast or Consumed Only Liquids at Least Once during the Week		*p*-Value	Children Who Consumed Well-Balanced Breakfast ≥ 5 Times per Week		Children Who Consumed Well-Balanced Breakfast < 5 Times per Week		*p*-Value
	*n*	%	*n*	%		*n*	%	*n*	%	
Sample size	155		45			117		83		
Sex girls boys	90 65	58.06% 41.94%	24 21	53.33% 46.67%	0.573	61 56	52.14% 47.86%	53 30	63.86% 36.14%	0.099
Mean age (years)	8.6		8.73		0.256	8.63		8.66		0.705

**Table 3:** Number (percentage) of children who consumed well-balanced breakfast every day on weekdays and weekend days.

Time Period	Yes	No	*p*-Value
School days	60 (30%)	86 (43%)	0.007
Weekend days	140 (70%)	114 (57%)	

**Table 4:** Content of selected food and beverage items in analyzed meals in group of children who consumed well-balanced breakfast 5 or more days per week or less than 5 days per week.

Variables	Children Who Consumed Well-Balanced Breakfast ≥ 5 Times per Week		Children Who Consumed Well-Balanced Breakfast < 5 Times per Week		*p*-Value
	*n*	%	*n*	%	
**Content of selected food items in meals**					
number of observations	805	100%	499	100%	
sandwich	509	63.23%	287	57.52%	0.040
sweet roll/bun	22	2.73%	21	4.21%	0.147
cereals or porridge	225	27.95%	80	16.03%	<0.001
yogurt	150	18.63%	82	16.43%	0.313
eggs	105	13.04%	70	14.03%	0.612
vegetables	204	25.24%	60	12.02%	<0.001
fruit	194	24.10%	85	17.03%	0.002
fruit or vegetables	361	44.84%	128	25.65%	<0.001
other	18	2.24%	22	4.41%	0.027
**Content of selected beverage items in meals**					
number of observations	817	100%	566	100%	
water	322	39.41%	149	26.33%	<0.001
tea	295	36.11%	203	35.87%	0.927
milk	275	33.66%	105	18.55%	<0.001
cacao	49	6%	81	14.31%	<0.001
fruit juice in a carton	115	14.08%	48	8.48%	0.002
fresh cold-pressed fruit juice	22	2.69%	25	4.42%	0.082
sweet drink	40	4.9%	32	5.65%	0.533
other	6	0.73%	12	2.12%	0.025

**Table 5:** Consumption frequency of selected food items for breakfast during the week in group of children who consumed well-balanced breakfast 5 or more days per week or less than 5 days per week.

Food Item	Children Who Consumed Well-Balanced Breakfast ≥ 5 Times per Week	Children Who Consumed Well-Balanced Breakfast < 5 Times per Week	*p*-Value
**sandwich**			0.006
M ±	4.35 ± 2.35	3.46 ± 2.09	
Me	5	3	
Min-Max	0–7	0–7	
**sweet roll/bun**			0.515
M ±	0.19 ± 0.68	0.25 ± 0.71	
Me	0	0	
Min-Max	0–5	0–4	
**cereals or porridge**			<0.001
M ±	1.92 ± 2.19	0.96 ± 1.58	
Me	1	0	
Min-Max	0–7	0–7	
**yogurt**			0.224
M ±	1.28 ± 1.89	0.99 ± 1.32	
Me	0	0	
Min-Max	0–7	0–7	
**eggs**			0.695
M ±	0.90 ± 0.97	0.84 ± 0.94	
Me	1	1	
Min-Max	0–3	0–4	
**vegetables**			<0.001
M ±	1.74 ± 1.86	0.72 ± 1.24	
Me	1	0	
Min-Max	0–7	0–5	
**fruit**			0.015
M ±	1.66 ± 2.01	1.02 ± 1.46	
Me	1	0	
Min-Max	0–7	0–5	
**fruit or vegetables**			<0.001
M ±	3.09 ± 2.33	1.54 ± 1.73	
Me	3	1	
Min-Max	0–7	0–6	

**Table 6:** Consumption frequency of selected beverage items for breakfast during the week in group of children who consumed well-balanced breakfast 5 or more days per week or less than 5 days per week.

Beverage Item	Children Who Consumed Well-Balanced Breakfast ≥ 5 Times per Week	Children Who Consumed Well-Balanced Breakfast < 5 Times per Week	*p*-Value
**water**			0.011
M ±	2.75 ± 2.73	1.80 ± 2.43	
Me	2	0	
Min-Max	0–7	0–7	
**tea**			0.841
M ±	2.52 ± 2.67	2.45 ± 2.55	
Me	2	2	
Min-Max	0–7	0–7	
**milk**			<0.001
M ±	2.35 ± 2.24	1.27 ±1.79	
Me	2	0	
Min-Max	0–7	0–7	
**cacao**			0.009
M ±	0.42 ± 0.98	0.98 ± 1.97	
Me	0	0	
Min-Max	0–7	0–7	
**juice**			0.073
M ±	0.98 ± 1.85	0.58 ± 1.04	
Me	0	0	
Min-Max	0–7	0–5	
**sweet drink**			0.804
M ±	0.34 ± 1.25	0.39 ± 1.20	
Me	0	0	
Min-Max	0–7	0–6	

## Data Availability

The data presented in this study are available on request from the corresponding author.

## Supplementary Materials

The following are available online at https://www.mdpi.com/article/10.3390/nu13072241/s1, Table S1: The classification of each food and drink item according to the scoring designed by Italian research group.

## References

[1] Affenito S.G. (2007). Breakfast: A Missed Opportunity. J. Am. Diet. Assoc..

[2] Rampersaud G.C. (2008). Benefits of Breakfast for Children and Adolescents: Update and Recommendations for Practitioners. Am. J. Lifestyle Med..

[3] Zipp A., Eissing G. (2019). Studies on the influence of breakfast on the mental performance of school children and adolescents. J. Public Health.

[4] Benton D., Jarvis M. (2007). The role of breakfast and a mid-morning snack on the ability of children to concentrate at school. Physiol. Behav..

[5] Pivik R.T., Tennal K.B., Chapman S.D., Gu Y. (2012). Eating breakfast enhances the efficiency of neural networks engaged during mental arithmetic in school-aged children. Physiol. Behav..

[6] Adolphus K., Lawton C.L., Dye L. (2013). The effects of breakfast on behaviour and academic performance in children and adolescents. Front. Hum. Neurosci..

[7] Szajewska H., Ruszczyński M. (2010). Systematic review demonstrating that breakfast consumption influences body weight outcomes in children and adolescents in Europe. Crit. Rev. Food Sci. Nutr..

[8] Silva F.A., Padez C., Sartorelli D.S., Oliveira R.M.S., Netto M.P., Mendes L.L., Candido A.P.C. (2018). Cross-sectional study showed that breakfast consumption was associated with demographic, clinical and biochemical factors in children and adolescents. Acta Paediatr..

[9] Wadolowska L., Hamulka J., Kowalkowska J., Ulewicz N., Gornicka M., Jeruszka-Bielak M., Kostecka M., Wawrzyniak A. (2019). Skipping breakfast and a meal at school: Its correlates in adiposity context. report from the ABC of healthy eating study of polish teenagers. Nutrients.

[10] Ardeshirlarijani E., Namazi N., Jabbari M., Zeinali M., Gerami H., Jalili R.B., Larijani B., Azadbakht L. (2019). The link between breakfast skipping and overweigh/obesity in children and adolescents: A meta-analysis of observational studies. J. Diabetes Metab. Disord..

[11] Liu J., Gibson D., Stearne K., Dobbin S.W. (2019). Skipping breakfast and non–high-density lipoprotein cholesterol level in school children: A preliminary analysis. Public Health.

[12] Deshmukh-Taskar P.R., Nicklas T.A., O’Neil C.E., Keast D.R., Radcliffe J.D., Cho S. (2010). The Relationship of Breakfast Skipping and Type of Breakfast Consumption with Nutrient Intake and Weight Status in Children and Adolescents: The National Health and Nutrition Examination Survey 1999–2006. J. Am. Diet. Assoc..

[13] Giménez-Legarre N., Flores-Barrantes P., Miguel-Berges M.L., Moreno L.A., Santaliestra-Pasías A.M. (2020). Breakfast Characteristics and Their Association with Energy, Macronutrients, and Food Intake in Children and Adolescents: A Systematic Review and Meta-Analysis. Nutrients.

[14] Inchley J., Currie D., Budisavljevic S., Torsheim T., Jåstad A., Cosma A. (2020). Spotlight on Adolescent Health and Well-Being: Findings from the 2017/2018 Health Behaviour in School-Aged Children (HBSC) Survey in Europe and Canada.

[15] Pedersen T.P., Holstein B.E., Flachs E.M., Rasmussen M. (2013). Meal frequencies in early adolescence predict meal frequencies in late adolescence and early adulthood. BMC Public Health.

[16] Lepicard E.M., Maillot M., Vieux F., Viltard M., Bonnet F. (2017). Quantitative and qualitative analysis of breakfast nutritional composition in French schoolchildren aged 9–11 years. J. Hum. Nutr. Diet..

[17] Bellisle F., Hébel P., Salmon-Legagneur A., Vieux F. (2018). Breakfast Consumption in French Children, Adolescents, and Adults: A Nationally Representative Cross-Sectional Survey Examined in the Context of the International Breakfast Research Initiative. Nutrients.

[18] Afeiche M.C., Taillie L.S., Hopkins S., Eldridge A.L., Popkin B.M. (2017). Breakfast Dietary Patterns among Mexican Children Are Related to Total-Day Diet Quality. J. Nutr..

[19] Tiso D., Baldini M., Piaggesi N., Ferrari P., Biagi P., Malaguti M., Lorenzini A. (2010). “7 days for my health”: A new tool to evaluate kids’ lifestyle. Agro. Food Ind. Hi Tech.

[20] Catalani F., Gibertoni D., Lorusso G., Rangone M., Dallolio L., Todelli S., Lorenzini A., Tiso D., Marini S., Leoni E. Consumo e adeguatezza della prima colazione durante l’arco di una settimana in un campione di bambini della scuola primaria. Proceedings of the 51 Congresso Nazionale Societa Italiana di Igiene Abstract Book.

[21] Healthy Eating Recommendations: Plate of Healthy Eating. https://ncez.pzh.gov.pl/abc-zywienia/talerz-zdrowego-zywienia/.

[22] Pyramid of Healthy Nutrition and Physical Activity for Children. http://www.izz.waw.pl/strona-gowna/3-aktualnoci/aktualnoci/643-piramida-zdrowego-zywienia-i-stylu-zycia-dzieci-i-mlodziezy.

[23] Fijałkowska A., Oblacińska A., Stalmach M. (2017). Nadwaga i otyłość u polskich 8-latków w świetle uwarunkowań biologicznych behawioralnych i społecznych. Raport z międzynarodowych badań WHO Childhood Obesity Surveillance Initiative (COSI). Overweight and Obesity among Polish 8-Year-Olds in the Context of Biological, Behavioural and Social Determinants.

[24] Zielińska M., Hamułka J., Gajda K. (2015). Family influences on breakfast frequency and quality among primary school pupils in Warsaw and its surrounding areas. Rocz. Panstw. Zakl. Hig..

[25] Galczak-Kondraciuk A., Stempel P., Czeczelewski J. (2018). Assessment of nutritional behaviours of children aged 7–12 attending to primary schools in Biala Podlaska, Poland. Rocz. Panstw. Zakl. Hig..

[26] Ostachowska-Gasior A., Piwowar M., Kwiatkowski J., Kasperczyk J., Skop-Lewandowska A. (2016). Breakfast and Other Meal Consumption in Adolescents from Southern Poland. Int. J. Environ. Res. Public Health.

[27] Papoutsou S., Briassoulis G., Hadjigeorgiou C., Savva S.C., Solea T., Hebestreit A., Pala V., Sieri S., Kourides Y., Kafatos A. (2014). The combination of daily breakfast consumption and optimal breakfast choices in childhood is an important public health message. Int. J. Food Sci. Nutr..

[28] Vissers P.A.J., Jones A.P., Corder K., Jennings A., Van Sluijs E.M.F., Welch A., Cassidy A., Griffin S. (2013). Breakfast consumption and daily physical activity in 9-10-year-old British children. Public Health Nutr..

[29] ALBashtawy M. (2017). Breakfast Eating Habits Among Schoolchildren. J. Pediatr. Nurs..

[30] Koca T., Akcam M., Serdaroglu F., Dereci S. (2017). Breakfast habits, dairy product consumption, physical activity, and their associations with body mass index in children aged 6–18. Eur. J. Pediatr..

[31] Ramotowska A., Szypowski W., Kunecka K., Szypowska A. (2017). Ocena czynników wpływających na konsumpcję śniadań wśród warszawskiej młodzieży w wieku szkolnym—rola w prewencji otyłości the assessment of the factors affecting breakfast habits in youth living in Warsaw, the role in obesity prevention. Pediatr. Endocrinol..

[32] Mullan B.A., Singh M. (2010). A systematic review of the quality, content, and context of breakfast consumption. Nutr. Food Sci..

[33] Alexy U., Wicher M., Kersting M. (2010). Breakfast trends in children and adolescents: Frequency and quality. Public Health Nutr..

[34] Raaijmakers L.G.M., Bessems K.M.H.H., Kremers S.P.J., Van Assema P. (2010). Breakfast consumption among children and adolescents in the Netherlands. Eur. J. Public Health.

[35] Rito A.I., Dinis A., Rascôa C., Maia A., de Carvalho Martins I., Santos M., Lima J., Mendes S., Padrão J., Stein-Novais C. (2019). Improving breakfast patterns of portuguese children—An evaluation of ready-to-eat cereals according to the European nutrient profile model. Eur. J. Clin. Nutr..

[36] Dye L. (2017). The Importance of Breakfast in Europe. A Review of National Policies and Health Campaigns.

