# Supplementary material for: Breakfast Frequency and Composition in a Group of Polish Children Aged 7–10 Years
Source: Nutrients. 2021 Jun 29;13(7):2241. doi: 10.3390/nu13072241 (PMC8308292; doi:10.3390/nu13072241)
Supplement: Supplementary file 1 [file nutrients-13-02241-s001.zip › nutrients-1258845-supplementary.pdf]

Supplementary Table S1. The classification of each food and drink item according to the scoring designed by the Italian research group.

Food or beverage item	Category / Content of selected nutrient or product					
	water	carbohydrates	protein or dairy product	fiber	vitamins and minerals	free sugars
water	1					
milk	1		1			
cacao	1		1			- 1
tea	1					
fruit juice	1				1	
cold-pressed fruit juice	1			1	1	
sweet beverage	1					- 1
sandwich with cheese or cottage cheese		1	1			
sandwich with ham		1	1			
sandwich with chocolate		1				- 1
sandwich with jam		1				- 1
cereals		1				
sweet roll/bun		1				- 1
yogurt		1	1			
eggs			1			
vegetables				1	1	
fruit				1	1	

This score was designed to use together with the diary ‘Seven days for my health’ by the Italian authors: F. Catalani, D. Gibertoni, G. Lorusso, M. Rangone, L. Dallolio, S. Todelli, A. Lorenzini, D. Tiso, S. Marini, and E. Leoni [20].
